# A Clamping Force Estimation Method Based on a Joint Torque Disturbance Observer Using PSO-BPNN for Cable-Driven Surgical Robot End-Effectors

**DOI:** 10.3390/s19235291

**Published:** 2019-12-01

**Authors:** Zhengyu Wang, Daoming Wang, Bing Chen, Lingtao Yu, Jun Qian, Bin Zi

**Affiliations:** 1 School of Mechanical Engineering, Hefei University of Technology, Hefei 230009, China; wangzhengyu_hfut@hfut.edu.cn (Z.W.); chbing@hfut.edu.cn (B.C.); qianjun@hfut.edu.cn (J.Q.); binzi@hfut.edu.cn (B.Z.); 2 Tianjin Key Laboratory of Aerospace Intelligent Equipment Technology, Tianjin Institute of Aerospace Mechanical and Electrical Equipment, Tianjin 300301, China; 3 College of Mechanical and Electrical Engineering, Harbin Engineering University, Harbin 150001, China; yulingtao@hrbeu.edu.cn

**Keywords:** surgical robot end-effector, clamping force estimation, joint torque disturbance observer, PSO-BPNN, cable tension measurement

## Abstract

The ability to sense external force is an important technique for force feedback, haptics and safe interaction control in minimally-invasive surgical robots (MISRs). Moreover, this ability plays a significant role in the restricting refined surgical operations. The wrist joints of surgical robot end-effectors are usually actuated by several long-distance wire cables. Its two forceps are each actuated by two cables. The scope of force sensing includes multidimensional external force and one-dimensional clamping force. This paper focuses on one-dimensional clamping force sensing method that do not require any internal force sensor integrated in the end-effector’s forceps. A new clamping force estimation method is proposed based on a joint torque disturbance observer (JTDO) for a cable-driven surgical robot end-effector. The JTDO essentially considers the variations in cable tension between the actual cable tension and the estimated cable tension using a Particle Swarm Optimization Back Propagation Neural Network (PSO-BPNN) under free motion. Furthermore, a clamping force estimator is proposed based on the forceps’ JTDO and their mechanical relations. According to comparative analyses in experimental studies, the detection resolutions of collision force and clamping force were 0.11 N. The experimental results verify the feasibility and effectiveness of the proposed clamping force sensing method.

## 1. Introduction

In recent years, more and more researchers, companies and hospitals have paid attention to the development, commercial aspects, and application of minimally-invasive surgical robot (MISR) techniques. The advantages of robot-assisted minimally-invasive surgery (MIS) include positioning accuracy, easier realization of MIS, an improved success rate, a reduction in pain, and a reduction in recovery time [1,2]. Many MISR systems including the da Vinci surgical system [3], the DLR MIRO [4], the Raven system [5] have been shown to be valid and feasible and to provide advancements in MIS operations. The capacity to sense force is an important technique for force feedback, haptics, and safe interaction control in MISRs. This ability can also help to restrict refined operations and improve operational safety [6]. Moreover, this ability can help surgeons to determine tissue hardness and evaluate the anatomical and histological properties of object organs in order to perform better manipulation in MIS [7,8]. Thus, the study of force sensing is an important research direction of MISR.

The wrist joints of a surgical robot end-effector usually have multiple degrees of freedom to ensure good flexibility and dexterity [9]. Cable-driven actuators have been widely used in rehabilitation and medical robots [10,11,12]. Furthermore, flexible continuum instruments present good application prospects. Hwang and Kwon proposed a novel constrained strong continuum manipulator by using auxiliary links attached to the main continuum links [13]. Li et al. developed a flexible endoscope based on the tendon-driven continuum mechanism [14]. The wrist joints of an end-effector are usually actuated by several long-distance cables with a small diameter. Each of its two forceps are actuated by two cables. The scope of force sensing includes multidimensional external force (including three-dimensional force and three-dimensional torque) and one-dimensional clamping force. Use of a surgical robot end-effector with the ability to sense force is an important and a feasible way to realize feedback from an external force or a clamping force.

Two methods for sensing external forces have been studied by MISR system researchers. One is the direct sensing method, which integrates force sensors into the surgical instrument’s tip. Some surgical tools have been designed for an MISR system with the ability to sense force [15,16,17]. Li et al. designed and developed a three-axis force sensor using a resistance-based sensing method [18]. Yu et al. developed a six-dimensional force/torque sensor with a double-crossbeam structure for a surgical robot end-effector [19]. Lim et al. proposed a kind of forceps with an optical fiber Bragg grating sensor integrated into it [20]. Kim et al. developed a surgical robot with a multi-axis force sensing instrument [21,22]. Radó et al. developed a surgery robot with a three-axis force sensor integrated into it to provide force feedback [21]. However, because wrist joints need to be designed to be narrow and small in size in order to meet the requirements of surgical robot end-effectors, and considering the limiting factors of the disinfection method, the material hemolysis effect, and the economic cost, many challenges and difficulties remain with the application of these force sensing methods by integrating internal force sensors into the tip of surgical forceps [23,24].

The other method for sensing external forces is the indirect sensing method, which uses system information including current, torque, tension, displacement, pressure, and a visual image of the laparoscope. The design of an external force disturbance observer or a similar estimation method has attracted a great deal of attention. Zhao and Nelson developed a cable-driven decoupled surgical robot end-effector and proposed a method for estimating the three-axis force using a motor current [25]. Because the friction and elasticity of cables are omitted in the overall model, as well as the filtering processing of the motor current, so the estimated accuracy was limited with relatively big errors. Li et al. [26] and Haraguchi et al. [27] both proposed external force disturbance observers for the force estimating the force acting at the forceps of pneumatically driven MISR. These studies were purely based on the system dynamic model and the cylinder pressure disturbance observers. The results showed good dynamic performance with acceptable estimated accuracy. Xue et al. proposed a tension sensor using fiber gratings for estimating the grasping force in a laparoscope surgical robot [28]. The property index of the sensor showed good performance. The hysteresis characteristics of the sensor and the friction between the cable and beams affect the resolution of the proposed sensor. They also proposed a method for estimating the grasping force based on a model of cable-pulley systems considering its tension transmission characteristics [29]. These two clamping force estimation methods were deeply affected by the model precision of cable-pulley transmission system. Li and Hannaford [30] proposed a Gaussian process regression for predicting the clamping force of a cable-driven surgical robot end-effector for the Raven system. The estimated accuracy was still the main problem for these clamping force estimation methods using the motor currents. In addition, artificial neural networks and deep learning algorithms provide us with another way to realize sensorless force sensing in MISRs [31,32,33]. Yu et al. proposed a bidimensional external force and clamping force sensing method based on changes in cable tension for a surgical micromanipulator [34]. The results showed acceptable accuracy. However, the comprehensive resistance of the cable tension was estimated and limited by the BP Neural network under free motion. This work was the preliminary study of the clamping force sensing compared with the new estimation method in this paper. Hwang and Lim proposed a force estimation method based on a deep learning method that utilize sequential images of an object’s shape changed by an external force [35]. It was difficult to learn and predict the interaction force using such many sequential and variational images in real time, due to the camera movement during the surgery operation. Huang et al. proposed a method for clamping force estimation based on a neural network for a cable-driven surgical robot [36]. The results showed the training process and training errors, but not considered the generalization ability verification. Marban et al. proposed a force estimation model for robotic surgery based on convolutional neural networks and long-short term memory networks [37]. The camera and organs were static while the surgical instrument was in motion. The real-time estimation is still a big challenge during the dynamic process of the real surgery.

To summarize, the property that these above proposed indirect sensing methods have in common is the combination or segregation of the disturbance observers and neural networks or learning methods. The accuracy, rapidity and robustness of the clamping force estimation are still the main challenges for the cable-driven surgical robot end-effector without the forceps’ internal force sensors. The motivation and contribution of this study are trying to find a new way of the clamping force estimation, which is considering the cable-driven system dynamics, the cable tension measurements and estimation, and the joint torque disturbance observer of the forceps in real time, such that aims to achieve a good comprehensive performance with low-cost and easy realization.

This paper focuses on one-dimensional clamping force sensing method that do not require any internal force sensors to be integrated into the wrist joints of a surgical robot end-effector. The main contribution of this study is the development of a novel method for estimating the clamping force of a forceps based on a joint torque disturbance observer, which essentially considers the variations in cable tension between the actual cable tension and the real-time estimated cable tension using a Particle Swarm Optimization Back Propagation Neural Network (PSO-BPNN) under free motion of the end-effector. A clamping force estimator is proposed based on the JTDO and the mechanical relations in the forceps. The main advantages of this method are the combination of cable-driven system dynamics and joint torque disturbance observer using PSO-BPNN, for improving the comprehensive performance. We verify the estimation method through a series of experiments with an equivalent experimental system.

## 2. Methods

### 2.1. Description of the 3-Degrees of Freedom (3-DoF) Cable-Driven Surgical Robot End-Effector

**Figure 1:**
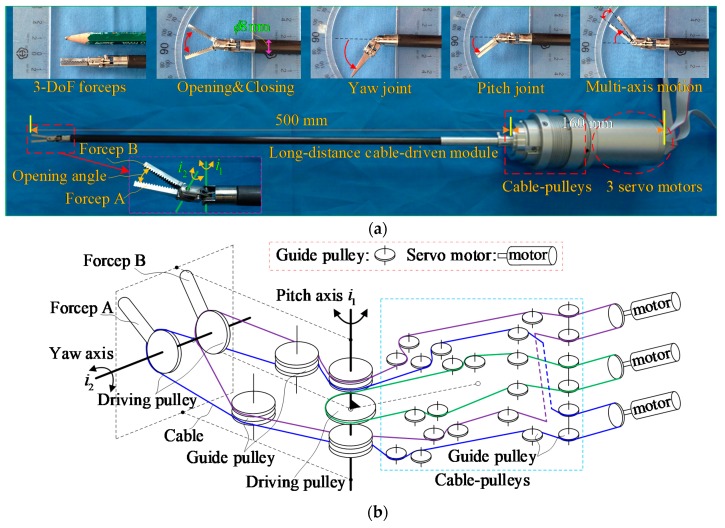
3-DoF cable-driven surgical robot end-effector. (**a**) Experimental prototype; (**b**) Principle diagram.

A surgical robot end-effector is a key device in a surgical robotic system. Figure 1a shows the experimental prototype of a 3-DoF surgical robot end-effector, which is actuated by three cable-driven actuators with six cables. The external diameter of the wrist joints and the slender shaft is limited to 8 mm.

Figure 1b shows the principle diagram of the configurational characteristics and the cable-driven systems. This 3-DoF forceps of a surgical robot end-effector has one yaw joint and two pitch joints, which are all actuated by cables. Meanwhile, the 3-DoF consist of yaw, pitch, and opening & closing. The opening & closing and pitch movement are compound motions between forceps A and B with different combinations of rotation directions. A multi-axis motion is the result of a 3-DoF compound action. The wrist joints of the end-effector are actuated by three long-distance cable-driven modules with a cable of approximately 500 mm in length inside of the slender shaft. The cable-pulley systems form the transition bridge between the wrist joints and three servo motors. Obviously, clamping force estimation focuses on the force conditions of forceps A and B for the 3-DoF cable-driven surgical robot end-effector. Furthermore, the driving torques of forceps A and B are directly related to the driving cable tensions. This means that the relationship between the driving torques and the cable tensions can be used to estimate the clamping force.

### 2.2. Equivalent Experimental System for the Surgical Robot End-Effector

In order to study external force and clamping force sensing methods, we designed and built an equivalent experimental prototype for the 3-DoF cable-driven surgical robot end-effector. 

**Figure 2:**
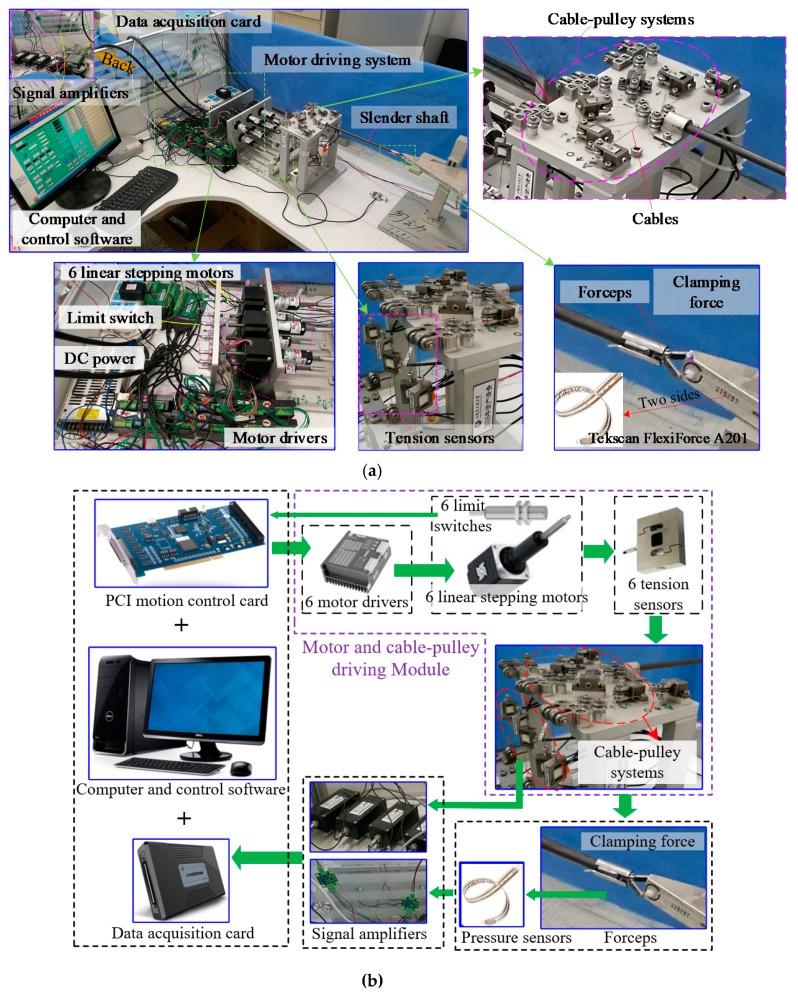
Experimental set-up. (**a**) Equivalent experimental prototype; (**b**) System composition block diagram.

As shown in Figure 2a, a clamping force sensing study can be performed by using this equivalent experimental system. The wrist joints are the same as shown in Figure 1. Their cable-pulley systems are expanded into a horizontal plate form, in order to achieve convenient installation and an easy layout. The equivalent experimental prototype consists of 3-DoF wrist joints, one slender shaft, cable-pulley systems, a cable tension detection module, motor driving system, a data acquisition system, a computer, and control software.

Overall, the difference between Figure 1 and Figure 2 are in the motor driving system. Figure 1 shows three servo motors for driving six cables. Figure 2 shows six linear stepping motors for driving six cables. Figure 2b shows a block diagram of the system’s composition. The nominal diameter of the driving wire cable is 0.45 mm. The measuring range of the tension sensor is 0–50 N. The max subdivs value of the linear stepping motor driver is 512. The motion control card can drive six motors. Six tension sensors are integrated between the motor driving system and the cable-pulley systems. This means that the cable tension of the input side can be measured. For detecting the actual clamping force, two pressure sensors (FlexiForce A201, Tekscan^®^, South Boston, MA, USA) are installed on the two sides of the triangular block, which is the grasping object for forceps A and B. In terms of overall function and robotic mechanisms, the equivalent experimental system (shown in Figure 2) is equal to the 3-DoF cable-driven surgical robot end-effector (shown in Figure 1).

As mentioned in the descriptions above, the equivalent experimental prototype is designed equivalent with the 3-DoF cable driven surgical robot end-effector. Their mechanical structures are the same on the aspect of mechanism principle. Moreover, they have the same 3-DoF wrist joints and forceps. The other main reason of equivalence principle is that, their kinematic mapping methods between the motor position and joint angle are the same principles only with different parameters. This means that the equivalent experimental prototype has the function and capability to represent the kinematics, dynamics, external forces, clamping force and kinematic mapping of the 3-DoF cable driven surgical robot end-effector. In the experiments on clamping force, the pitch joint lacked convenience. The deeply reason is that the manufacturing and assembly errors of the pitch joint greatly influence the decoupling of the wrist joints. Therefore, in order to verify the feasibility and effectiveness of the clamping force sensing method that we proposed in this paper, we discarded the pitch joint as shown in Figure 2. This means that the experiments of clamping force estimation were carried out with a pitch joint angle of 0 degrees and a specific opening angle of 65 degrees, which is a limitation of the experimental mechanical structure.

### 2.3. Strategy for Estimating the Clamping Force of the Surgical Robot End-Effector

#### 2.3.1. Modeling the System Dynamics of the Forceps

**Figure 3:**
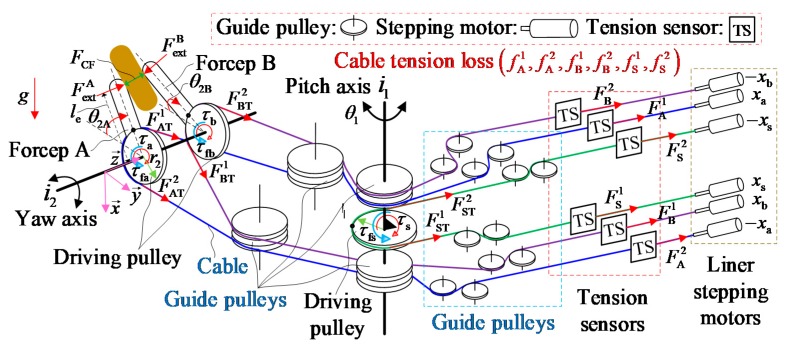
A simplified dynamic model of the cable-driven surgical robot end-effector. linear.

As mentioned in previous sections, the mechanical model between the joint torques and the cable tensions is the key to establishing the overall dynamics model, which provides a model for calculating the clamping force. Figure 3 shows a simplified dynamic model of the cable-driven surgical robot end-effector. Because the initial cable tensions were preadjusted to suitable values during the adjustment process, the elasticity of cables can be neglected in the modeling process. Moreover, the friction of the cable-pulley system is temporarily hided in the cable tension losses $F_{f}$ of the overall dynamics, as well as the nonlinear characteristics of cables. These two factors are the uncertain models. But they can be estimated by the PSO-BPNN. In order to study the problem of estimating the clamping force of the cable-driven surgical robot end-effector, the modelling, analysis and experiment were all carried out at the system’s zero position without considering the coupling problem and manufacturing errors of the wrist joints’ motion. The main reason is that the experiment condition is limited at the system’s zero position according to the measurement of the clamping force using two flexible pressure sensors. Under these circumstances, the feasibility and effectiveness of the estimation method can be verified.

The subscripts A/a, B/b, S/s in the following formulas denote the relations to yaw joint A, yaw joint B, and pitch joint S, respectively. $F_{A}^{1}$, $F_{B}^{1}$, and $F_{S}^{1}$ denote the respective actual measured values of the motor driving cables. $F_{A}^{2}$, $F_{B}^{2}$, and $F_{S}^{2}$ denote the respective actual measured values of the motor returning cables. $F_{\mathrm{AT}}^{1}$, $F_{\mathrm{BT}}^{1}$, and $F_{\mathrm{ST}}^{1}$ denote the respective driving cable tensions. $F_{\mathrm{AT}}^{2}$, $F_{\mathrm{BT}}^{2}$, and $F_{\mathrm{ST}}^{2}$ denote the respective returning cable tensions.$f_{A}^{1}$, $f_{B}^{1}$, and $f_{S}^{1}$ denote the respective driving cable tension losses. $f_{A}^{2}$, $f_{B}^{2}$, and $f_{S}^{2}$ denote the respective returning cable tension losses. The cable tension losses include the frictions of the cable-pulley systems, the nonlinear characteristics of cables, and other uncertain items. $\tau_{a}$, $\tau_{b}$, and $\tau_{s}$ denote the respective joint driving torques. $\tau_{\mathrm{fa}}$, $\tau_{\mathrm{fb}}$, and $\tau_{\mathrm{fs}}$ denote the respective joint friction torques. $x_{a}$, $x_{b}$, and $x_{s}$ denote the respective motor displacements of driving cables. $-x_{a}$, $-x_{b}$, and $-x_{s}$ denote the respective motor displacements of returning cables. $\theta_{2A}$, $\theta_{2B}$, and $\theta_{1}$ denote the respective joint angles of yaw joint A, yaw joint B and pitch joint S. Meanwhile, the opening angle of forceps A and B can be calculated as $\left(\theta_{2A}+\theta_{2B}\right)$, according to the initial position in Figure 2. $r_{1}$ and $r_{2}$ denote the effective drive radius of yaw joint A, yaw joint B and pitch joint S, respectively.

Suppose that $F_{M}$ is the set of actual measured values of the motor driving cable tensions for forceps:

(1) $$F_{M}={\left[\begin{array}{cccc} F_{A}^{1} & F_{A}^{2} & F_{B}^{1} & F_{B}^{2} \end{array}\right]}^{T}$$

Suppose that $F_{T}$ is the set of cable tensions for actuating forceps A and B:

(2) $$F_{T}={\left[\begin{array}{cccc} F_{\mathrm{AT}}^{1} & F_{\mathrm{AT}}^{2} & F_{\mathrm{BT}}^{1} & F_{\mathrm{BT}}^{2} \end{array}\right]}^{T}$$

Suppose that $F_{f}$ is the set of cable tension losses for actuating forceps A and B:

(3) $$F_{f}={\left[\begin{array}{cccc} f_{A}^{1} & f_{A}^{2} & f_{B}^{1} & f_{B}^{2} \end{array}\right]}^{T}$$

Obviously, from Equations (1) to (3), the following relationship can be obtained:

(4) $$F_{M}=F_{T}+F_{f}$$

The driving torques $\tau_{\mathrm{ab}}$ of forceps A and B can be calculated as:

(5) $$\tau_{\mathrm{ab}}=\left[\begin{array}{c} \tau_{a}\\ \tau_{b} \end{array}\right]=r_{2}\left[\begin{array}{c} F_{\mathrm{AT}}^{1}-F_{\mathrm{AT}}^{2}\\ F_{\mathrm{BT}}^{1}-F_{\mathrm{BT}}^{2} \end{array}\right]=r_{2}\left[\begin{array}{c} \left(F_{A}^{1}-f_{A}^{1}\right)-\left(F_{A}^{2}+f_{A}^{2}\right)\\ \left(F_{B}^{1}-f_{B}^{1}\right)-\left(F_{B}^{2}+f_{B}^{2}\right) \end{array}\right]$$

where, $\tau_{a}$ and $\tau_{b}$ denote the driving torques of forceps A and B. 

Suppose that ${\hat{F}}_{E}$ is the set of estimated values from the cable tension sensors under free motion of the forceps, which means that no clamping force or external force is applied to the wrist joints of the surgical robot end-effector. An artificial neural network model can be employed for the estimated values of the cable tension sensors’ tensions ${\hat{F}}_{E}$ under free motion. Suppose that ${\hat{F}}_{\mathrm{fE}}$ is the set of cable tension losses for actuating forceps A and B under free motion:

(6) $${\hat{F}}_{E}={\left[\begin{array}{cccc} {\hat{F}}_{A}^{1} & {\hat{F}}_{A}^{2} & {\hat{F}}_{B}^{1} & {\hat{F}}_{B}^{2} \end{array}\right]}^{T}$$

(7) $${\hat{F}}_{\mathrm{fE}}={\left[\begin{array}{cccc} {\hat{f}}_{\mathrm{AE}}^{1} & {\hat{f}}_{\mathrm{AE}}^{2} & {\hat{f}}_{\mathrm{BE}}^{1} & {\hat{f}}_{\mathrm{BE}}^{2} \end{array}\right]}^{T}$$

Therefore, the set of cable tension disturbances from the tension sensor measurement modules can be defined as ${\hat{F}}_{D}$:

(8) $${\hat{F}}_{D}={\left[\begin{array}{cccc} {\hat{F}}_{\mathrm{AD}}^{1} & {\hat{F}}_{\mathrm{AD}}^{2} & {\hat{F}}_{\mathrm{BD}}^{1} & {\hat{F}}_{\mathrm{BD}}^{2} \end{array}\right]}^{T}=F_{M}-{\hat{F}}_{E}$$

The simplified dynamic model of forceps A and B can be given as:

(9) $$\begin{array}{ll} \tau_{\mathrm{ab}} & =\left[\begin{array}{c} \tau_{a}\\ \tau_{b} \end{array}\right]=\left[\begin{array}{c} J_{A}{\ddot{\theta }}_{2A}+g(\theta_{2A})+\tau_{\mathrm{fa}}+F_{\mathrm{ext}}^{A}l_{\mathrm{eA}}\\ J_{B}{\ddot{\theta }}_{2B}+g(\theta_{2B})+\tau_{\mathrm{fb}}+F_{\mathrm{ext}}^{B}l_{\mathrm{eB}} \end{array}\right]\\ & =r_{2}\left[\begin{array}{c} F_{\mathrm{AT}}^{1}-F_{\mathrm{AT}}^{2}\\ F_{\mathrm{BT}}^{1}-F_{\mathrm{BT}}^{2} \end{array}\right]\\ & =r_{2}\left[\begin{array}{c} \left(F_{A}^{1}-F_{A}^{2}\right)-\left(f_{A}^{1}+f_{A}^{2}\right)\\ \left(F_{B}^{1}-F_{B}^{2}\right)-\left(f_{B}^{1}+f_{B}^{2}\right) \end{array}\right] \end{array}$$

where, $J_{A}$ and $J_{B}$, $\tau_{\mathrm{fa}}$ and $\tau_{\mathrm{fb}}$, $\theta_{2A}=x_{a}/r_{2}$ and $\theta_{2B}=x_{b}/r_{2}$, $l_{\mathrm{eA}}$ and $l_{\mathrm{eB}}$, and $F_{\mathrm{ext}}^{A}$ and $F_{\mathrm{ext}}^{B}$ are the equivalent rotating inertias, the joint frictions, the joint angles, the arms of external force, and the external force of the joints of forceps A and B, respectively. We neglected the forceps’ gravities since they are installed in the horizontal plane.

Equation (9) can be revised as Equation (10) when there is no external force or clamping force under free motion:

(10) $$\begin{array}{ll} {\hat{\tau }}_{\mathrm{ab}} & =\left[\begin{array}{c} {\hat{\tau }}_{a}\\ {\hat{\tau }}_{b} \end{array}\right]=\left[\begin{array}{c} J_{A}{\ddot{\theta }}_{2A}+g(\theta_{2A})+{\hat{\tau }}_{\mathrm{fa}}\\ J_{B}{\ddot{\theta }}_{2B}+g(\theta_{2B})+{\hat{\tau }}_{\mathrm{fb}} \end{array}\right]\\ & =r_{2}\left[\begin{array}{c} \left({\hat{F}}_{A}^{1}-{\hat{F}}_{A}^{2}\right)-\left({\hat{f}}_{\mathrm{AE}}^{1}+{\hat{f}}_{\mathrm{AE}}^{2}\right)\\ \left({\hat{F}}_{B}^{1}-{\hat{F}}_{B}^{2}\right)-\left({\hat{f}}_{\mathrm{BE}}^{1}+{\hat{f}}_{\mathrm{BE}}^{2}\right) \end{array}\right] \end{array}$$

Considering that linear stepping motors were chosen to be the drive units for the wrist joints, the acceleration and deceleration modes were set to the ‘S’ type in the motion control card, their acceleration and deceleration time were set to be 0.02 s, and the constant velocity was set to be variable. Because the inertias of the wrist joints are very small, their inertial forces only have marginal effects on the wrist joints’ system dynamics, so the inertia forces of the wrist joints were ignored in this paper. If the joints’ driving torques can be estimated under free motion, then Equations (9) and (10) can be simplified as Equations (11) and (12), respectively:

(11) $$\begin{array}{ll} \tau_{\mathrm{ab}} & =\left[\begin{array}{c} \tau_{a}\\ \tau_{b} \end{array}\right]=\left[\begin{array}{c} \tau_{\mathrm{fa}}+F_{\mathrm{ext}}^{A}l_{\mathrm{eA}}\\ \tau_{\mathrm{fb}}+F_{\mathrm{ext}}^{B}l_{\mathrm{eB}} \end{array}\right]\\ & =r_{2}\left[\begin{array}{c} \left(F_{A}^{1}-F_{A}^{2}\right)-\left(f_{A}^{1}+f_{A}^{2}\right)\\ \left(F_{B}^{1}-F_{B}^{2}\right)-\left(f_{B}^{1}+f_{B}^{2}\right) \end{array}\right] \end{array}$$

(12) $$\begin{array}{ll} {\hat{\tau }}_{\mathrm{ab}} & =\left[\begin{array}{c} {\hat{\tau }}_{a}\\ {\hat{\tau }}_{b} \end{array}\right]=\left[\begin{array}{c} {\hat{\tau }}_{\mathrm{fa}}\\ {\hat{\tau }}_{\mathrm{fb}} \end{array}\right]\\ & =r_{2}\left[\begin{array}{c} \left({\hat{F}}_{A}^{1}-{\hat{F}}_{A}^{2}\right)-\left({\hat{f}}_{\mathrm{AE}}^{1}+{\hat{f}}_{\mathrm{AE}}^{2}\right)\\ \left({\hat{F}}_{B}^{1}-{\hat{F}}_{B}^{2}\right)-\left({\hat{f}}_{\mathrm{BE}}^{1}+{\hat{f}}_{\mathrm{BE}}^{2}\right) \end{array}\right] \end{array}$$

Combining Equations (11) and (12), the joint torque disturbance ${\hat{\tau }}_{D}={\left[\begin{array}{cc} {\hat{\tau }}_{\mathrm{aD}} & {\hat{\tau }}_{\mathrm{bD}} \end{array}\right]}^{T}$ of forceps A and B can be given as:

(13) $$\begin{array}{ll} {\hat{\tau }}_{D} & =\tau_{\mathrm{ab}}-{\hat{\tau }}_{\mathrm{ab}}=\left[\begin{array}{c} \tau_{\mathrm{fa}}-{\hat{\tau }}_{\mathrm{fa}}+F_{\mathrm{ext}}^{A}l_{\mathrm{eA}}\\ \tau_{\mathrm{fb}}-{\hat{\tau }}_{\mathrm{fb}}+F_{\mathrm{ext}}^{B}l_{\mathrm{eB}} \end{array}\right]\\ & =r_{2}\left[\begin{array}{c} \left({\hat{F}}_{\mathrm{AD}}^{1}-{\hat{F}}_{\mathrm{AD}}^{2}\right)-\left(f_{A}^{1}+f_{A}^{2}\right)+\left({\hat{f}}_{\mathrm{AE}}^{1}+{\hat{f}}_{\mathrm{AE}}^{2}\right)\\ \left({\hat{F}}_{\mathrm{BD}}^{1}-{\hat{F}}_{\mathrm{BD}}^{2}\right)-\left(f_{B}^{1}+f_{B}^{2}\right)+\left({\hat{f}}_{\mathrm{BE}}^{1}+{\hat{f}}_{\mathrm{BE}}^{2}\right) \end{array}\right] \end{array}$$

Under the circumstances of free motion and no clamping force, the joint frictions and cable tension losses of forceps A and B satisfy the condition approximated below:

(14) $${\left[\begin{array}{cccc} \left(f_{A}^{1}+f_{A}^{2}\right) & \left(f_{B}^{1}+f_{B}^{2}\right) & \tau_{\mathrm{fa}} & \tau_{\mathrm{fb}} \end{array}\right]}^{T}\approx {\left[\begin{array}{cccc} \left({\hat{f}}_{A}^{1}+{\hat{f}}_{A}^{2}\right) & \left({\hat{f}}_{B}^{1}+{\hat{f}}_{B}^{2}\right) & {\hat{\tau }}_{\mathrm{fa}} & {\hat{\tau }}_{\mathrm{fb}} \end{array}\right]}^{T}$$

Therefore, the joint torque disturbance ${\hat{\tau }}_{D}$ of Equation (13) can be further simplified as:

(15) $${\hat{\tau }}_{D}=\left[\begin{array}{c} {\hat{\tau }}_{\mathrm{aD}}\\ {\hat{\tau }}_{\mathrm{bD}} \end{array}\right]\approx r_{2}\left[\begin{array}{c} {\hat{F}}_{\mathrm{AD}}^{1}-{\hat{F}}_{\mathrm{AD}}^{2}\\ {\hat{F}}_{\mathrm{BD}}^{1}-{\hat{F}}_{\mathrm{BD}}^{2} \end{array}\right]=\left[\begin{array}{c} F_{\mathrm{ext}}^{A}l_{\mathrm{eA}}\\ F_{\mathrm{ext}}^{B}l_{\mathrm{eB}} \end{array}\right]$$

Furthermore, the estimated external force ${\hat{F}}_{\mathrm{ext}}$ of forceps A and B can be given by Equation (16):

(16) $${\hat{F}}_{\mathrm{ext}}={\left[\begin{array}{cc} {\hat{F}}_{\mathrm{ext}}^{A} & {\hat{F}}_{\mathrm{ext}}^{B} \end{array}\right]}^{T}\approx {\left[\begin{array}{cc} \frac{{\hat{\tau }}_{\mathrm{aD}}}{l_{\mathrm{eA}}} & \frac{{\hat{\tau }}_{\mathrm{bD}}}{l_{\mathrm{eB}}} \end{array}\right]}^{T}$$

Finally, the estimated clamping force of forceps A and B can be calculated by Equation (17):

(17) $${\hat{F}}_{\mathrm{CF}}\approx \frac{1}{2}\left(\left|{\hat{F}}_{\mathrm{ext}}^{A}\right|+\left|{\hat{F}}_{\mathrm{ext}}^{B}\right|\right)$$

#### 2.3.2. Strategy for Estimating the Clamping Force

**Figure 4:**
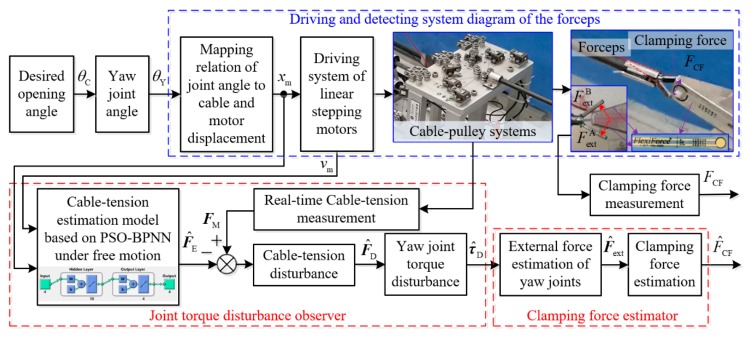
Strategy for estimating the clamping force of the forceps.

According to the process for modeling the forceps’ clamping force, a strategy for estimating the clamping force of the forceps is given as shown in Figure 4. Essentially, the block diagram of this estimation strategy is a kind of open-loop form, which is constructed according to the open-loop control strategy of the joints’ motion using the feedback of the kinematic mapping relation.

Firstly, the desired opening angle or grasping angle $\theta_{C}$ is input into the control software; then, it is converted to yaw joint angle $\theta_{Y}$ according to the kinematic relation.

Secondly, angle $\theta_{Y}$ is input into the driving system according to the mapping relation of the joint angle to the cable and motor displacement; then, the calculated motor displacement $x_{m}$ and setting motor velocity $v_{m}$ are input into the driving system of the linear stepping motors.

Thirdly, the motors pull or push the cable loops with the desired displacement while the force sensors measure the driving and returning cable tensions $F_{M}$. Meanwhile, the wrist joints are actuated by the difference in the cable tensions in the cable-pulley systems. Forceps A and B are actuated by the difference in tension with the result of an opening or a closing action on the two flexible pressure sensors, which provide comparisons of the measured joint external forces $F_{\mathrm{ext}}^{A}$ and $F_{\mathrm{ext}}^{B}$, and the measured clamping force $F_{\mathrm{CF}}$.

Fourthly, the motor displacement $x_{m}$ and the motor velocity $v_{m}$ of forceps A and B are input into the cable tension estimation model based on PSO-BPNN under free motion in order to estimate the tensions ${\hat{F}}_{E}$. Therefore, the cable tension disturbance ${\hat{F}}_{D}$ can be calculated by inputting the actual cable tensions $F_{M}$ and the estimated cable tensions ${\hat{F}}_{E}$. The yaw joint torque disturbance ${\hat{\tau }}_{D}$ can be obtained using the cable tension disturbance ${\hat{F}}_{D}$.

Finally, the external force ${\hat{F}}_{\mathrm{ext}}$ of the yaw joints can be estimated by Equation (16), and the clamping force ${\hat{F}}_{\mathrm{CF}}$ can be calculated using the clamping force estimator. To sum up, the strategy for estimating the clamping force is based on the measured motor driving cable tensions, the cable tension model based on PSO-BPNN under free motion, the joint torque disturbance observer, and the clamping force estimator that does not require internal force sensors to be integrated into the end tips of the wrist joints. 

#### 2.3.3. Joint Torque Disturbance Observer Using PSO-BPNN

The purpose of designing the yaw joint torque disturbance observer shown in Equation (15) is to estimate or predict the variation in the motor’s driving cable tension as compared with the same motor’s displacement and velocity under free motion. This means that the forceps’ external force or clamping force affects the motor’s driving cable tension with respect to the condition of free motion or no clamping force. The core requirement for this joint torque disturbance observer is to build a high-accuracy model for predicting the cable tension under free motion with the same motor displacement and velocity. The parameters estimations with evolutionary algorithms using tweezers show its superiority in a coupled nonlinear dynamics [38]. In this paper, the PSO-BPNN was employed to fit an artificial neural network model for estimating or predicting the motor’s driving cable tension under free motion.

The artificial neural networks can approximate any function to an arbitrary degree of accuracy due to its high learning capability and parallel computing nature [39]. The BPNN is a supervised artificial neural network, and it is widely used for nonlinear and non-convex function approximation [40]. However, its main disadvantages include slow learning speed, a propensity to easily fall into a local minimum, a limited number of network layers, and overfitting [41]. The traditional BPNN can be improved by global optimization of PSO to solve the problems of oscillation, slow convergence and local extremum in the training process [42]. 

In this paper, the PSO-BPNN is employed to fit an artificial neural network model for estimating or predicting the motor driving cable tension under free motion. PSO-BPNN [43] is a combination of Particle Swarm Optimization and a BP neural network. Because the PSO algorithm is based on a heuristic learning algorithm, it can search different regions of the solution space at the same time, avoid falling into local minima and realize global optimization. In each iteration of the optimal solution, the particle updates itself by tracking two extreme values. The first one is called individual extreme value, which is the optimal solution found by the particle itself. The other one is called global extreme value, which is the optimal solution found by the whole population. When the two optimal extreme values are found, the particle updates its velocity and position according to the following formula [44]:

(18) $$\left\{ \begin{array}{l} v_{id}=wv_{id}+c_{1}u_{1}\left(p_{id}-x_{id}\right)+c_{2}u_{2}\left(p_{gd}-x_{id}\right)\\ x_{id}=x_{id}+v_{id} \end{array}\right.$$

where, $x_{id}$ and $v_{id}$ are the position and velocity of the *i*-th particle, respectively; $p_{id}$ is the optimal position that the *i*-th particle searches for; $p_{gd}$ is the optimal position searched by the whole particle swarm; $c_{1}$ and $c_{2}$ are learning factors; $u_{1}$ and $u_{2}$ are uniform random numbers within [−1,1]; w is the inertia weight.

The PSO algorithm was used to train the BP neural network, and the weight and threshold of each neuron were taken to be a particle’s iterative optimization of the solution space. The specific steps for PSO of the BP neural network algorithm are introduced in [45,46]. The specific steps for PSO of the BP neural network algorithm are as follows:

(a) Determine the topological structure of the BP neural network and set the number of neurons in each layer of the BP neural network. The particle population is initialized, and the velocity and position of each particle are randomly set. The main operating parameters of particle swarm optimization are shown in Table 1.
(b) Calculate the fitness value Fit (i) of each particle;
(c) Compare the fitness value Fit (i) of each particle with the individual extreme value. If Fit (i) > pbest (i), replace pbest (i) with Fit (i).
(d) Compare the fitness value Fit (i) of each particle with the global extreme value gbest (i). If Fit (i) > gbest (i), replace gbest (i) with Fit (i).
(e) Update the position and velocity of each particle according to Equation (18);
(f) If the condition is satisfied (the error is sufficiently small or the number of cycles has reached its maximum), exit; otherwise, return to the second step (b);
(g) The global extreme value gbest (i) from the PSO algorithm is used as the weight and threshold for the BP neural network and to train the neural network with training samples;
(h) The generalization ability of the PSO-BPNN can be tested by simulation with the test samples.

To sum up, the PSO-BPNN plays an important role in the online estimation of motor cable tensions under free motion. The estimated values are input into the joint torque disturbance observer as contrasting values. The clamping force estimator can be established on this basis.

#### 2.3.4. The Clamping Force Estimator

As shown in Figure 4 and described in Equations (15) and (16), the external forces ${\hat{F}}_{\mathrm{ext}}$ of forceps A and B can be estimated using the joint torque disturbance observer and the contact force arm. Equation (17) shows the definition and an estimation of the comprehensive clamping force ${\hat{F}}_{\mathrm{CF}}$ of the two forceps A and B. The clamping force estimator proposed in this paper essentially considers and combines the system’s dynamic characteristics and an artificial neural network. Moreover, the general accuracy of the trained PSO-BPNN model determines the accuracy of the method for estimating the clamping force.

## 3. Results and Discussion

In order to validate and evaluate the comprehensive performance of the proposed method for estimating clamping force based on a joint torque disturbance observer using PSO-BPNN, a series of experiments were carried out. In this section, we provide the results from the cable tension estimation model based on the PSO-BPNN under free motion, and the experimental results of clamping force estimation considering collision detection and a clamping action.

### 3.1. Training and Testing Results from the Cable Tension Estimation Model Based on the PSO-BPNN under Free Motion

A series of experiments were carried out to explore the potential relations between the motor’s displacement and velocity and the measured cable tension value under free motion. The displacement corresponding to one pulse of the linear stepping motor is 0.00127 mm. The acceleration and deceleration modes are selected as “S” type in the motion control card. The acceleration and deceleration time are set to 0.02s, and the constant motion velocity is set to be variable. Five kinds of motors’ velocities (0.5, 0.6, 0.7, 0.8 and 1.0 mm/s) with increasing ladder are planned for the joint motion with the forceps’ opening angle ranging from zero to maximum angle. The maximum opening angle of the forceps A and B is 130 degrees. This means that forceps A and B are actuated with joint angle range [0,65] degrees on the opposite direction, respectively. The input data were the displacement and velocity of the two linear stepping motors for pulling the driving cables of forceps A and B. The output data were the cable tension values that were measured through four tension sensors. Table 1 shows the main parameters of the PSO-BPNN. 

The training data comprised 90% of the randomized input data and their related output data. The remaining 10% of the randomized input and output data were used as the testing data for examining the generalization ability and prediction accuracy of the PSO-BPNN. The number of training data was 4892, and the number of testing data was 544.

**Figure 5:**
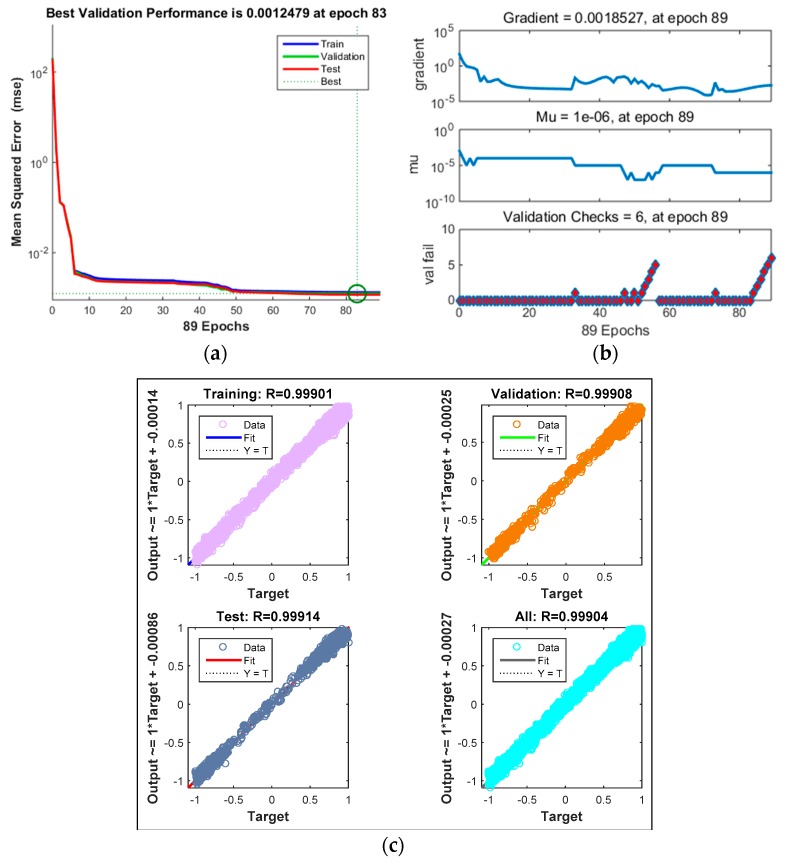
The training and testing outcome indicators of the PSO-BPNN model. (**a**) Mean squared error; (**b**) Gradient, mu and validation checks; (**c**) Regression results.

**Figure 6:**
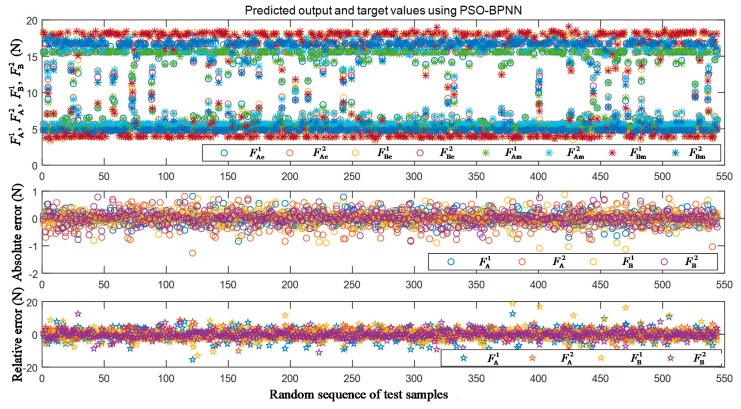
The estimation results of the testing data using the PSO-BPNN model.

The training and testing outcome indicators of the PSO-BPNN model are shown as Figure 5. We can see that the trained PSO-BPNN model has good comprehensive performance. Figure 6 shows the estimation results when the testing data were input into the PSO-BPNN model. The mean square errors of the testing data were 0.2171, 0.2918, 0.2459, and 0.2379 N with respect to the estimation ability of ${\hat{F}}_{E}$, which are the motor cable tensions for actuating forceps A and B under free motion.

### 3.2. Experimental Results of Clamping Force Estimation

**Figure 7:**
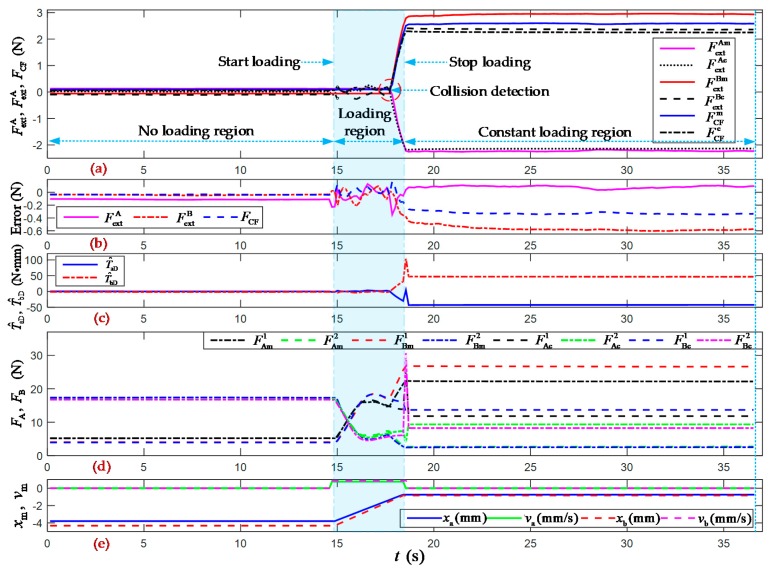
Experimental results of the continuous clamping force estimation with a collision analysis. (**a**) Measured and estimated values of the external forces and clamping force of forceps A and B. The superscripts *m* and *e* denote the measured and estimated values, respectively; (**b**) Errors in the estimated external forces and clamping force; (**c**) Joint torque disturbances of forceps A and B; (**d**) Measured and estimated values of the cable tensions of forceps A and B. The superscripts *m* and *e* denote the measured and estimated values, respectively; (**e**) Displacements and velocities of the linear stepping motors for pulling the driving cables of forceps A and B.

**Figure 8:**
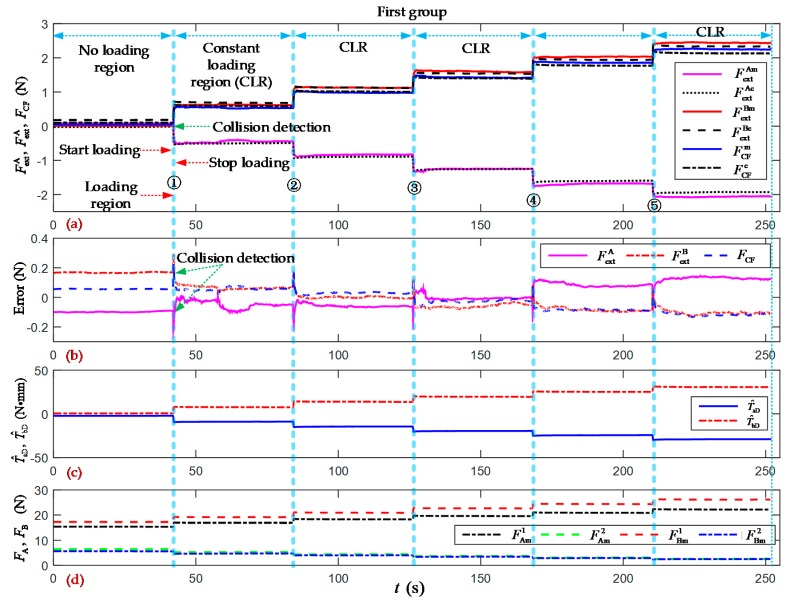
Experimental results of the incremental clamping force estimation (first group). (**a**) Measured and estimated values of the external forces and clamping force of forceps A and B. The superscripts *m* and *e* denote the measured and estimated values; (**b**) Errors in the estimated external forces and clamping force; (**c**) Joint torque disturbances of forceps A and B; (**d**) Measured values of the cable tensions of forceps A and B. The superscript *m* denotes the measured values.

**Figure 9:**
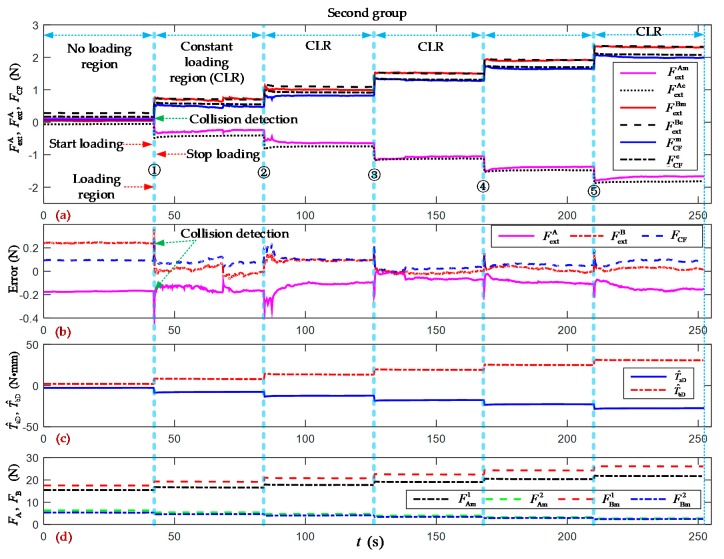
Experimental results of the incremental clamping force estimation (second group). (**a**) Measured and estimated values of the external forces and clamping force of forceps A and B. The superscripts *m* and *e* denote the measured and estimated values, respectively; (**b**) Errors in the estimated external forces and clamping force; (**c**) Joint torque disturbances of forceps A and B; (**d**) Measured values of the cable tensions of forceps A and B. The superscript *m* denotes the measured values.

As shown in Figure 2a and Figure 4, the two forceps A and B were actuated from an opening angle of 120 degrees to the clamping position limited by the contact object. To test the comprehensive performance of the proposed method for clamping force estimation, the continuous and stair- loading types of clamping motion experiments were carried out, as shown in Figure 7, Figure 8 and Figure 9. “No loading region” means that the forceps perform the clamping motion without contacting the object. “Start loading” means that the forceps come into contact with the object for the first time. “Stop loading” means that the forceps come into contact with the object for the final time. “Loading region” means that the clamping force increases the area between the start and stop loading moments. “Collision detection” means that the clamping force can be estimated with a set threshold value, which is called the collision detection resolution. “Constant loading region” means that the clamping motion had stopped due to constant external forces and the clamping force of forceps A and B.

Figure 7 shows the experimental results of the continuous clamping force estimation with a collision analysis. The collision detection threshold was 0.11 N for the estimated external forces of forceps A and B. Over the whole region, the overall root mean square errors (RMSEs) of the estimated external forces were 0.1010 N and 0.4035 N for forceps A and B, respectively; and the overall RMSE of the clamping force was 0.2321 N. The overall average errors in the estimated external forces were –0.0088 N and –0.3042 N for forceps A and B, respectively; the overall average error in the estimated clamping force was –0.1751 N; and the errors in the estimated clamping force can be summarized in the interval of [–0.3482, 0.1718] N. In the constant loading region, the estimation accuracies of the external forces ${\hat{F}}_{\mathrm{ext}}^{A}$ and ${\hat{F}}_{\mathrm{ext}}^{B}$ and the clamping force ${\hat{F}}_{\mathrm{CF}}$ were 93.83%, 80.13%, and 86.91%, respectively.

Figure 8 and Figure 9 show the two groups of experimental results of the incremental clamping force estimation. At the end of the no loading region, five stepwise incremental loading motions were performed in the clamping force experiments. The first contact position was set at the opening angle (65 degrees). The estimated cable tensions ${\hat{F}}_{A}^{1}$, ${\hat{F}}_{A}^{2}$, ${\hat{F}}_{B}^{1}$, and ${\hat{F}}_{B}^{2}$ from the trained PSO-BPNN model were 14.8259 N, 6.7541 N, 17.1017 N, and 5.7025 N, respectively. The estimated external forces ${\hat{F}}_{\mathrm{ext}}^{A}$ and ${\hat{F}}_{\mathrm{ext}}^{B}$ are compensated for with their overall average errors of –0.1395 N and –0.1404 N, respectively, from the first group.

Over the whole region of the first group of experiments as shown in Figure 8, the overall RMSE of the estimated external forces ${\hat{F}}_{\mathrm{ext}}^{A}$ and ${\hat{F}}_{\mathrm{ext}}^{B}$ were 0.0825 N and 0.0956 N, respectively; and the overall RMSE of the clamping force ${\hat{F}}_{\mathrm{CF}}$ was 0.070 N. The overall average errors in the estimated external forces were 0.00002 N and –0.00004 N, respectively. The overall average error in the estimated clamping force was –0.013 N, and the errors in the estimated clamping force can be summarized in the interval of [–0.1343, 0.2256] N.

Over the whole region of the second group of experiments as shown in Figure 9, the overall RMSE of the estimated external forces ${\hat{F}}_{\mathrm{ext}}^{A}$ and ${\hat{F}}_{\mathrm{ext}}^{B}$ was 0.1316 N and 0.1087 N, respectively. The overall RMSE of the clamping force ${\hat{F}}_{\mathrm{CF}}$ was 0.080 N. The overall average errors in the estimated external forces were –0.1223 N and 0.0628 N, respectively; and the overall average error in the estimated clamping force was 0.0736 N. The errors in the estimated clamping force can be summarized in the interval of [–0.0100, 0.2794] N.

### 3.3. Analysis and Discussion

When training a PSO-BPNN model, the estimation accuracy of the trained neural network can be limited by multiple effects, including the model parameter settings and the training data size. Because the five different velocities of the yaw joint motion were quantitative, the estimation accuracy of the trained PSO-BPNN model was only affected by the training data size. So, the estimation accuracy of the trained PSO-BPNN model could be increased by collecting more training data with a higher number of velocity bands. However, our trained PSO-BPNN model showed sufficiently good comprehensive performance to be employed in the estimation of the cable tension of forceps A and B.

Figure 7, Figure 8 and Figure 9 show the results of the clamping force estimation in the continuous and stair-loading types of clamping motion experiments. In the curves of the estimation errors and the joint torque disturbance, some relatively large errors arise at the beginning of the start loading region and the end of the stop loading region. The reasons for these errors include, on the one hand, the effects of ignoring the nonlinear elasticity of and the creep and hysteresis in the wire cables. These nonlinear parameters and factors cannot be totally included in the system dynamic model and PSO-BPNN model. On the other hand, the measured errors in the two flexible pressure sensors cannot be ignored when they are pressed at the beginning and at the end of the clamping action.

To sum up, the collision detection threshold was found to reach 0.11 N, and the clamping force estimation resolution was found to be the same as the collision detection threshold. The estimation accuracy for the static clamping force was greater than 86%. In this study, the proposed method for clamping force estimation was shown to perform well. The two groups of incremental clamping force estimation experiments demonstrated the more complete comprehensive performance of the proposed method, with average errors in the interval of [–0.0722, 0.2525] N. Meanwhile, the experiments showed that this method can provide effectively detection range in the interval of [0,2] N. Moreover, the length of forceps direct influences the arms of external force, this means that the effectively detection range can be extended by reducing the overall length of the forceps within reasonable limits. Overall, the experimental results mean that the proposed method for clamping force estimation has the potential to be used in the cable-driven end-effector of a surgical robotic system for MIS. 

## 4. Conclusions

In this study, we proposed a method for clamping force estimation based on a joint torque disturbance observer using PSO-BPNN for a cable-driven surgical robot end-effector. This estimation method considers both the cable-driven end-effector’s system dynamics and the estimated cable tension using the PSO-BPNN under free motion. Moreover, the clamping force can be estimated by only using known information about the motor’s displacement and velocity, and the measured cable tension value, without the need for internal force sensors to be integrated into the wrist joints of the surgical robot end-effector. The PSO-BPNN-based joint torque disturbance observer performed well in the disturbance estimation. The experimental results showed that the proposed method for clamping force estimation has good comprehensive performance. Our future work will focus on solving the decoupling precision problem in multi-DoF wrist joints, and the engineering and application of this method.

## Figures and Tables

**Table 1:** The main parameters of PSO-BPNN.

Parameters	Values
Swarm size	10
$c_{1}$, $c_{2}$	1.49445
Max iteration	30
w	1
Number of the input layer nodes	4
Number of the hidden layer nodes	10
Number of the output layer nodes	4
Number of neural network training	100
Learning rate of neural network	0.005
Percentage of training data	90%
Percentage of testing data	10%

## References

[1] Marcus H., Nandi D., Darzi A., Yang G. (2013). Surgical Robotics Through a Keyhole: From Today’s Translational Barriers to Tomorrow’s ‘Disappearing’ Robots. IEEE Trans. Bio-Med. Eng..

[2] Wang Z., Zi B., Ding H., You W., Yu L. (2018). Hybrid grey prediction model-based autotracking algorithm for the laparoscopic visual window of surgical robot. Mech. Mach. Theory.

[3] Mohareri O., Ramezani M., Adebar T., Abolmaesumi P., Salcudean S. (2013). Automatic Localization of the da Vinci Surgical Instrument Tips in 3-D Transrectal Ultrasound. IEEE Trans. Bio-Med. Eng..

[4] Blake H., Jacob R., Diana W., King H., Roan P., Cheng L., Glozman D., Ma J., Kosari S., White L. (2013). Raven-II: An Open Platform for Surgical Robotics Research. IEEE Trans. Bio-Med. Eng..

[5] Hagn U., Nickl M., Jörg S., Passig G., Bahls T., Nothhelfer A., Hacker F., Le-Tien L., Albu-Schäffer A., Konietschke R. (2008). The DLR MIRO: A versatile lightweight robot for surgical applications. Ind. Robot.

[6] Kim U., Lee D.H., Yoon W.J., Hannaford B., Choi H.R. (2015). Force sensor integrated surgical forceps for minimally invasive robotic surgery. IEEE Trans. Robot..

[7] Gonenc B., Chamani A., Handa J., Gehlbach P., Taylor R., Iordachita I. (2017). 3-DOF Force-Sensing Motorized Micro-Forceps for Robot-Assisted Vitreoretinal Surgery. IEEE Sens. J..

[8] Aviles A., Alsaleh S., Hahn J., Casals A. (2016). Towards retrieving force feedback in robotic-assisted surgery: A supervised neuro-recurrent-vision approach. IEEE Trans. Haptics.

[9] Wang Z., Zi B., Wang D., Qian J., You W., Yu L. (2019). External force self-sensing based on cable-tension disturbance observer for surgical instrument. IEEE Sens. J..

[10] Chen B., Zi B., Wang Z., Qin L., Liao W. (2019). Knee exoskeletons for gait rehabilitation and human performance augmentation: A state-of-the-art. Mech. Mach. Theory.

[11] Chen Q., Zi B., Sun Z., Li Y., Xu Q. (2019). Design and Development of a New Cable-Driven Parallel Robot for Waist Rehabilitation. IEEE/ASME Trans. Mech..

[12] Wu L., Crawford R., Roberts J. (2016). Dexterity analysis of three 6-DOF continuum robots combining concentric tube mechanisms and cable-driven mechanisms. IEEE Robot. Autom. Lett..

[13] Hwang M., Kwon D.S. (2019). Strong continuum manipulator for flexible endoscopic surgery. IEEE/ASME Trans. Mech..

[14] Ma X., Song C., Chiu P.W., Li Z. (2019). Autonomous Flexible Endoscope for Minimally Invasive Surgery with Enhanced Safety. IEEE Robot. Autom. Lett..

[15] Kuebler B., Seibold U., Hirzinger G. (2006). Development of actuated and sensor integrated forceps for minimally invasive surgery. Int. J. Med. Robot. Comp..

[16] Trejos A., Escoto A., Naish M., Patel R. (2017). Design and Evaluation of a Sterilizable Force Sensing Instrument for Minimally Invasive Surgery. IEEE Sens. J..

[17] Yu L., Yan Y., Yu X., Xia Y. (2018). Design and Realization of Forceps With 3-D Force Sensing Capability for Robot-Assisted Surgical System. IEEE Sens. J..

[18] Li K., Pan B., Zhan J., Gao W., Fu Y., Wang S. (2015). Design and performance evaluation of a 3-axis force sensor for MIS palpation. Sens. Rev..

[19] Yu H., Jiang J., Xie L., Liu L., Shi Y., Cai P. (2014). Design and static calibration of a six-dimensional force/torque sensor for minimally invasive surgery. Minim. Invasive Ther. Allied Technol..

[20] Lim S., Lee H., Park J. (2014). Grip force measurement of forceps with fibre Bragg grating sensors. Electron. Lett..

[21] Kim U., Lee D., Kim Y., Seok D.Y., So J., Choi H. (2017). S-surge: Novel portable surgical robot with multiaxis force-sensing capability for minimally invasive surgery. IEEE/ASME Trans. Mech..

[22] Kim U., Kim Y.B., So J., Seok D.Y., Choi H.R. (2018). Sensorized surgical forceps for robotic-assisted minimally invasive surgery. IEEE Trans. Ind. Electron..

[23] Radó J., Dücső C., Földesy P., Szebényi G., Nawrat Z., Rohr K., Fürjes P. (2018). 3D force sensors for laparoscopic surgery tool. Microsyst. Technol..

[24] Overtoom E., Horeman T., Jansen F., Dankelman J., Schreuder H. (2019). Haptic feedback, force feedback, and force-sensing in simulation training for laparoscopy: A systematic overview. J. Surg. Educ..

[25] Zhao B., Nelson C. (2016). Estimating Tool-Tissue Forces Using a 3-Degree-of-Freedom Robotic Surgical Tool. J. Mech. Robot..

[26] Li H., Kawashima K., Tadano K., Ganguly S., Nakano S. (2013). Achieving haptic perception in forceps’ manipulator using pneumatic artificial muscle. IEEE/ASME Trans. Mech..

[27] Haraguchi D., Kanno T., Tadano K., Kawashima K. (2015). A pneumatically driven surgical manipulator with a flexible distal joint capable of force sensing. IEEE/ASME Trans. Mech..

[28] Xue R., Ren B., Huang J., Yan Z., Du Z. (2018). Design and Evaluation of FBG-Based Tension Sensor in Laparoscope Surgical Robots. Sensors.

[29] Xue R., Du Z., Yan Z., Ren B. (2019). An estimation method of grasping force for laparoscope surgical robot based on the model of a cable-pulley system. Mech. Mach. Theory.

[30] Li Y., Hannaford B. (2017). Gaussian process regression for sensorless grip force estimation of cable-driven elongated surgical instruments. IEEE Robot. Autom. Lett..

[31] Liang Y., Du Z., Wang W., Yan Z., Sun L. (2019). An improved scheme for eliminating the coupled motion of surgical instruments used in laparoscopic surgical robots. Robot. Auton. Syst..

[32] Gessert N., Beringhoff J., Otte C., Schlaefer A. (2018). Force estimation from OCT volumes using 3D CNNs. Int. J. Comput. Ass. Rad..

[33] Li X., Cao L., Tiong A., Phan P., Phee S. (2019). Distal-end force prediction of tendon-sheath mechanisms for flexible endoscopic surgical robots using deep learning. Mech. Mach. Theory.

[34] Yu L., Wang W., Zhang F. (2018). External force sensing based on cable tension changes in minimally invasive surgical micromanipulators. IEEE Access.

[35] Hwang W., Lim S.C. (2017). Inferring Interaction Force from Visual Information without Using Physical Force Sensors. Sensors.

[36] Huang J., Yan Z., Xue R. Grip Force Estimation of Laparoscope Surgical Robot based on Neural Network Optimized by Genetic Algorithm. Proceedings of the 3rd International Conference on Robotics, Control and Automation.

[37] Marban A., Srinivasan V., Samek W., Fernández J., Casals A. (2019). A recurrent convolutional neural network approach for sensorless force estimation in robotic surgery. Biomed. Signal Proces..

[38] Verotti M., Di Giamberardino P., Belfiore N.P., Giannini O. (2019). A genetic algorithm-based method for the mechanical characterization of biosamples using a MEMS microgripper: Numerical simulations. J. Mech. Behav. Biomed..

[39] Yeh W.C. (2016). A squeezed artificial neural network for the symbolic network reliability functions of binary-state networks. IEEE Trans. Neural Netw. Learn. Syst..

[40] Jethmalani C.R., Simon S.P., Sundareswaran K., Nayak P.S.R., Padhy N.P. (2016). Auxiliary hybrid PSO-BPNN-based transmission system loss estimation in generation scheduling. IEEE Trans. Ind. Inform..

[41] Liu Q., Brigham K., Rao N.S. (2017). Estimation and fusion for tracking over long-haul links using artificial neural networks. IEEE Trans. Signal Inf. Process. Netw..

[42] Hou C., Yu X., Cao Y., Lai C., Cao Y. (2017). Prediction of synchronous closing time of permanent magnetic actuator for vacuum circuit breaker based on PSO-BP. IEEE Trans. Dielectr. Electr. Insul..

[43] Liu C., Ding W., Li Z., Yang C. (2017). Prediction of high-speed grinding temperature of titanium matrix composites using BP neural network based on PSO algorithm. Int. J. Adv. Manuf. Technol..

[44] Zhang C., Chen Z., Mei Q., Duan J. (2017). Application of particle swarm optimization combined with response surface methodology to transverse flux permanent magnet motor optimization. IEEE Trans. Magn..

[45] Ren C., An N., Wang J., Li L., Hu B., Shang D. (2014). Optimal parameters selection for BP neural network based on particle swarm optimization: A case study of wind speed forecasting. Knowl. Based syst..

[46] Mohamad E., Armaghani D., Momeni E., Yazdavar A., Ebrahimi M. (2018). Rock strength estimation: A PSO-based BP approach. Neural Comput. Appl..

